# Development and internal validation of a task-conditioned multimodal radiology–pathology model for breast cancer staging and biomarker profiling: a retrospective cohort study

**DOI:** 10.3389/fcell.2026.1863777

**Published:** 2026-08-21

**Authors:** Yang Liu, Yunfei Dong, Guiyun Zhang, Yue Wu, Shanshan Zhang, Yufo Chen, Ruirui Cao, Rui Wang, Chengliang Yin, Yumei Li

**Affiliations:** 1 Department of Medical Oncology, The First Affiliated Hospital of Bengbu Medical University, Bengbu, Anhui, China; 2 Faculty of Computing, Harbin Institute of Technology, Harbin, Heilongjiang, China; 3 Department of Biomedical Engineering, Faculty of Engineering, Universiti Malaya, Kuala Lumpur, Malaysia

**Keywords:** biomarker profiling, breast cancer, multimodal artificial intelligence, pathology, radiology

## Abstract

**Objectives:**

To retrospectively develop and evaluate a task-conditioned multimodal artificial intelligence framework, in which a learned task embedding and modality-availability mask generate endpoint-specific weights over predefined radiology-only, pathology-only, and fusion experts, for breast cancer staging and biomarker profiling.

**Methods:**

We retrospectively assembled a cohort of 923 patients with paired radiology and pathology data. Nine tasks included TNM components [tumor extent (T), nodal involvement (N), and distant metastasis (M)], clinical stage, histological grade, and estrogen receptor, progesterone receptor, HER2, and Ki-67 status. Five convolutional neural network backbones were benchmarked for each modality. Task-specific models were selected by validation AUROC, and a late-fusion transformer was used for paired data. We further evaluated learned MoE gating, task-conditioned MoE routing with task embeddings and modality-availability masks, missing-modality robustness, and paired-bootstrap AUROC comparisons. An independent external cohort assessed feasibility for mammography-based pathological complete response and pathology-based triple-negative breast cancer (TNBC) prediction.

**Results:**

Best single-modality models achieved AUROCs ranging from 0.606 to 0.990 across tasks, with an AUROC of 0.990 observed for M staging and strong performance observed for histological grade (AUROC, 0.949; accuracy, 0.908) and HER2 status (AUROC, 0.810; accuracy, 0.762). Fixed late fusion improved the mean AUROC from 0.813 to 0.826. Learned MoE gating and task-conditioned MoE further improved mean AUROC to 0.830 and 0.834, respectively. Task-conditioned MoE improved mean AUROC by 0.009 over fixed late fusion (95% CI, 0.005–0.013; P = 0.011) and by 0.021 over the best single-modality expert. Under 70% random missing-modality simulation, task-conditioned MoE outperformed fixed fusion with zero imputation by 0.067 mean AUROC. In external feasibility analyses, the mammography-based pCR model achieved an AUROC of 0.758, and the pathology-based triple-negative breast cancer model achieved an AUROC of 0.872.

**Conclusion:**

OmniBreast demonstrated the feasibility of task-conditioned radiology-only, pathology-only, and fused prediction in the internal nine-task benchmark. The external analyses provided preliminary support for feasibility on two related endpoint-extension tasks but did not establish generalizability across the nine primary tasks; multicenter external validation and prospective clinical evaluation remain necessary.

## 1 Introduction

Breast cancer management relies on the coordinated interpretation of imaging, pathology, and biomarker information. Imaging supports lesion detection and assessment of disease extent, pathological examination confirms tumor type and grade, and estrogen receptor (ER), progesterone receptor (PR), human epidermal growth factor receptor 2 (HER2), and Ki-67 guide systemic treatment and prognostic evaluation (Sung et al., 2021; Thomssen et al., 2021; Wolff et al., 2018). In routine practice, however, these data are often reviewed in separate reports and at different time points, which can limit integrated patient-level reasoning.

Artificial intelligence (AI) has shown value in breast imaging for cancer detection, risk prediction, and subtype characterization (Zhu et al., 2021; Gjesvik et al., 2024; Jiang et al., 2025; Schopf et al., 2024; Eisemann et al., 2025; Zhan et al., 2023; Carriero et al., 2025; Liu T. et al., 2022). Multimodal AI studies have further suggested that integrating imaging, pathology, and clinical variables can improve prediction of treatment response or recurrence risk (Lyu et al., 2025; Nishizawa et al., 2025; Zhang et al., 2025; Su et al., 2025; Keogan et al., 2025; Mbaye et al., 2025). In parallel, computational pathology models have been used to infer histological grade, receptor status, proliferation status, and slide-level risk directly from histological images (Akbarnejad et al., 2025; Høibø et al., 2025; Bae et al., 2023; Vorontsov et al., 2024; Lu et al., 2024; Xu et al., 2024; Ding et al., 2025). Medical vision-language models have also advanced through contrastive pretraining, medical knowledge augmentation, and image-text representation learning, and they are increasingly being explored for radiology reporting and outcome prediction (Wang et al., 2022; Tiu et al., 2022; Bannur et al., 2023; Wu et al., 2023; Shu et al., 2024; Tanno et al., 2025; Zhong et al., 2025). Despite these advances, many existing systems remain endpoint-specific, depend on a fixed combination of input modalities, and do not provide a flexible mechanism for switching among staging and biomarker questions in the same patient.

The central clinical and technical problem is therefore not only how to fuse radiology and pathology data, but also when fusion is useful and when a single modality is sufficient. For some endpoints, one modality may already provide adequate predictive signal, whereas other endpoints may benefit from complementary cross-modal information. A practical breast cancer AI framework should therefore route available patient data to task-appropriate models, avoid unnecessary computational burden, and indicate task-specific limitations when input evidence is incomplete. This motivates a task-adaptive framework rather than a single monolithic predictor. In this study, “task-conditioned” refers to a bounded expert-routing mechanism in which a learned embedding of the requested clinical endpoint, together with a mask indicating the available input modalities, is used to assign endpoint-specific weights to predefined radiology-only, pathology-only, and fusion experts.

**FIGURE 1:**
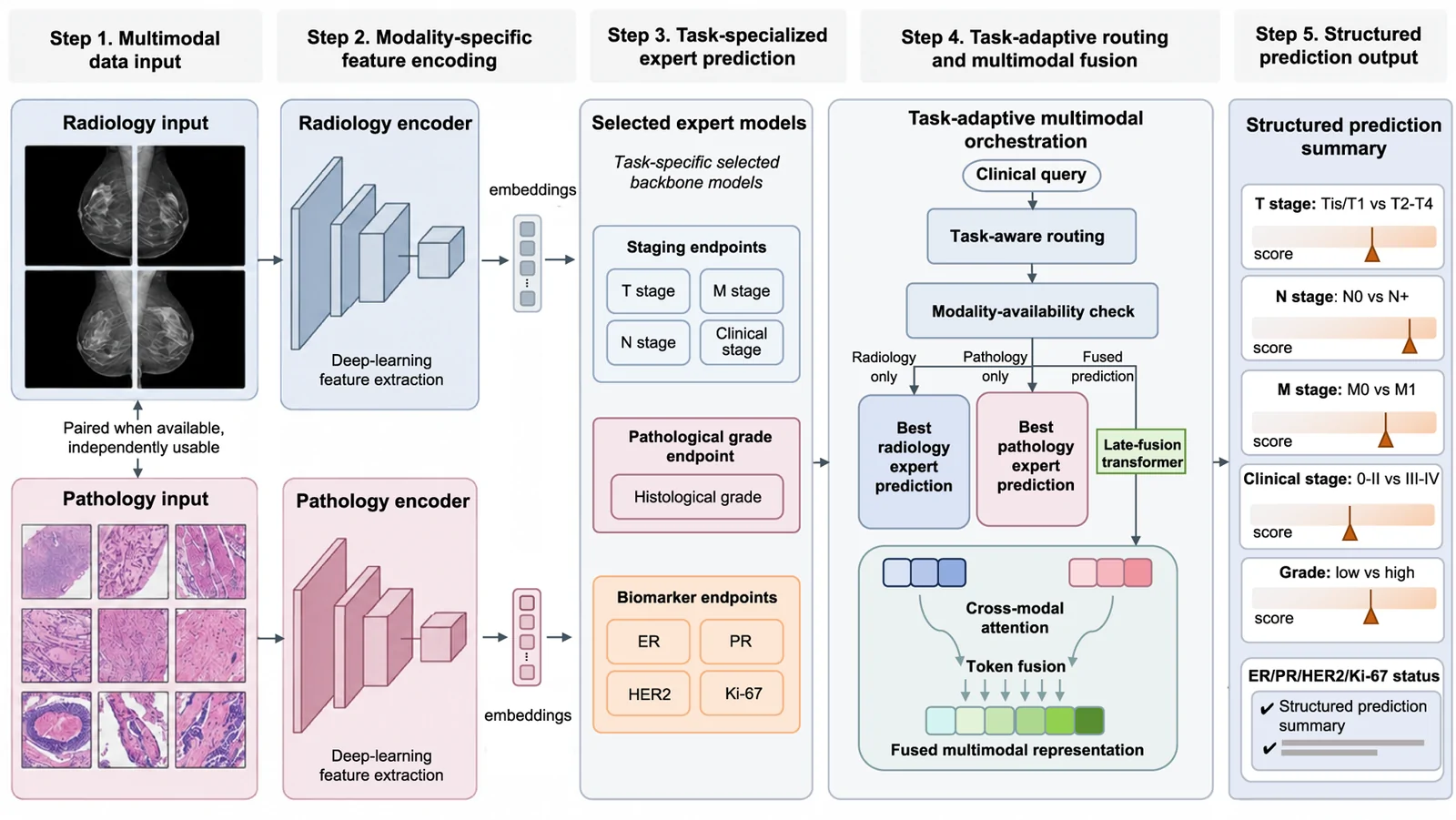
Overview of OmniBreast, a task-conditioned multimodal framework for breast cancer staging and biomarker profiling. Schematic of the OmniBreast pipeline, showing modality-specific encoders for radiology and pathology, task-specialized classifiers for nine clinically relevant endpoints (T/N/M staging, clinical stage, histological grade, ER/PR/HER2 status and Ki-67), and a late-fusion transformer that integrates modality embeddings when both modalities are available. The framework supports deterministic modality-availability routing as well as learned task-conditioned MoE routing, and aggregates calibrated probabilities and discrete predictions into a structured report for downstream decision support.

In this study, we developed OmniBreast, a task-conditioned multimodal radiology-pathology framework for breast cancer staging and biomarker profiling. The contribution of this work is fourfold: first, we evaluated a unified nine-task benchmark covering TNM staging, clinical stage, histological grade, and key biomarkers; second, we combined task-specific backbone selection with a lightweight late-fusion transformer to integrate complementary modality embeddings; third, we compared the fixed fusion workflow with learned dynamic routing baselines, including learned MoE gating and task-conditioned MoE routing with task embeddings and modality-availability masks; and fourth, we assessed subgroup consistency, efficiency, *post hoc* explainability, missing-modality robustness, and two external endpoint-extension tasks. We hypothesized that late multimodal fusion and task-conditioned dynamic routing would provide modest but consistent improvement over the strongest single-modality experts, particularly for tasks in which radiology and pathology carry complementary information. The workflow of the OmniBreast framework is illustrated in Figure 1.

## 2 Materials and methods

### 2.1 Study design, cohort and task definitions

We developed OmniBreast, a task-conditioned multimodal framework that combines task-specific deep models across paired radiology and pathology data to support integrated staging and biomarker profiling. The retrospective cohort comprised 923 patients with pretreatment mammographic images and matched routine hematoxylin and eosin–stained pathological slides. Clinical staging and biomarker labels were abstracted from finalized clinical and pathology records and were used solely as reference standards. All images included in the primary analyses were acquired before systemic treatment. To reflect clinically actionable decisions, we formulated nine supervised tasks: TNM staging components, including tumor extent (T; Tis/T1 vs T2–T4), nodal involvement (N; N0 vs N+), and distant metastasis (M; M0 vs M1), as well as clinical stage (0–II vs III–IV), histological grade (low vs high), and biomarker status for ER, PR, HER2, and Ki-67 (all binarized as negative vs positive/high according to report-defined criteria). All experiments were performed with patient-level splits to prevent leakage across tasks, and model selection was conducted within the training/validation partition; primary analyses were conducted on the internal cohort. In addition, two endpoint-extension experiments, namely, mammography-based pathological complete response (pCR) prediction and pathology-based triple-negative breast cancer (TNBC) prediction, were further evaluated in an independent external cohort from Hunan Cancer Hospital. To promote reproducibility and reduce *ad hoc* tuning, we followed a standardized, self-configuring workflow for preprocessing and training schedules, conceptually aligned with best practices from automated medical imaging pipelines (e.g., nnU-Net-style rule-driven configuration) (Isensee et al., 2021).

### 2.2 Data preprocessing and multimodal representation

The radiology input consisted exclusively of pretreatment full-field digital mammograms acquired in the standard craniocaudal and mediolateral oblique views. Mammograms were acquired using systems from Hologic, GE Healthcare, and Siemens Healthineers, accounting for approximately 46%, 34%, and 20% of examinations, respectively. No systemic staging examinations, including CT, PET/CT, bone scintigraphy, or other imaging used to determine distant metastatic status, were included. Radiology reports, pathology reports, TNM variables, treatment information, and other report-derived clinical data were not provided to the image models. Original DICOM images were de-identified, converted to single-channel arrays, cropped to the breast region, normalized using percentile-based intensity scaling between the 1st and 99th percentiles, and resized to 512 × 512 pixels.

The pathology input consisted exclusively of routine hematoxylin and eosin–stained whole-slide images; immunohistochemistry-stained slides for ER, PR, HER2, and Ki-67 were not included as model inputs. Slides were digitized using Leica Aperio AT2 scanners at ×40 magnification, corresponding to approximately 0.25 μm per pixel, and were analyzed at a 20× equivalent resolution. Tissue regions were identified using Otsu thresholding followed by morphological removal of small background components. Non-overlapping tiles of 224 × 224 pixels were extracted, and tiles containing less than 60% tissue were excluded. During training, 128 tiles were randomly sampled per patient at each epoch; during validation and testing, 128 tiles were selected using deterministic uniform sampling across the tissue mask. Tile-level embeddings were aggregated into a patient-level pathology representation using gated attention-based multiple-instance pooling. RandAugment was applied only during training, whereas no stochastic augmentation was used during validation or testing. All preprocessing, augmentation, and training procedures were implemented using the Medical Open Network for AI framework (Cubuk et al., 2020; Cardoso et al., 2022).

### 2.3 Model development, multimodal fusion, dynamic routing and evaluation

For each label, we trained five convolutional neural network (CNN) backbone baselines (ResNet, DenseNet, EfficientNet, RegNet, MobileNetV3) under matched training budgets, then selected the best-performing backbone for that task using validation AUROC. This best-backbone strategy served as the deterministic single-modality expert selection step for each endpoint and modality.

To construct fixed multimodal predictors, we implemented a late-fusion transformer that consumes modality-specific embeddings and learns cross-modal interactions via attention. The fusion design drew on hierarchical and windowed attention principles used in modern vision transformers (e.g., Swin Transformer) (Liu et al., 2021) while retaining a lightweight, deployment-oriented footprint. As an efficiency-oriented reference point for backbone modernization, we also considered design insights from contemporary ConvNet re-parameterizations (ConvNeXt) when configuring optimization and regularization schedules (Liu Z. et al., 2022). All convolutional encoders were initialized using ImageNet-pretrained weights. Models were optimized using AdamW with an initial learning rate of 1 × 10^−4^, a weight decay of 1 × 10^−4^, and a cosine-annealing learning-rate schedule with a five-epoch linear warm-up. Radiology models were trained with a batch size of 16, pathology models with a batch size of four patient-level tile bags, and the fixed-fusion and MoE models with a batch size of eight patients. Training was performed for a maximum of 100 epochs, with early stopping if validation AUROC did not improve for 15 consecutive epochs. Gradient clipping was applied at a maximum norm of 5.0. Experiments were repeated using random seeds 42, 3407, and 2024, and the checkpoint with the highest validation AUROC was retained for held-out testing. For each endpoint, the operating threshold was selected exclusively in the internal validation set by maximizing the Youden index and was subsequently fixed before evaluation of the held-out test set.

To directly compare OmniBreast with dynamic routing and mixture-of-experts baselines, we further implemented two learned routing variants. The learned MoE gating model combined the radiology-only, pathology-only, and fixed-fusion expert probabilities using a small multilayer-perceptron gate that produced modality-level expert weights. The task-conditioned MoE further incorporated a task embedding and a modality-availability mask, allowing the gate to learn task-specific and missing-modality-aware expert weights. The final probability was calculated as the weighted combination of the available expert outputs. We also evaluated two ablations by removing the task embedding or the modality mask, and included an oracle selector as a non-deployable upper bound that uses test-label information only to estimate the maximal attainable gain from per-case expert selection.

The radiology-only and pathology-only experts were trained independently using their corresponding modalities, whereas the fixed late-fusion model was trained using patients with paired radiology and pathology data. During task-conditioned MoE training, stochastic modality dropout was applied with a probability of 0.30, whereby either the radiology or pathology input was randomly masked while retaining at least one available modality. A binary modality-availability mask indicated whether radiology, pathology, or both modalities were present and was provided to the MoE gate together with the task embedding. The primary held-out evaluation used the originally available modalities without artificial masking. Missing-modality robustness was evaluated separately by randomly masking one modality in 30%, 50%, and 70% of held-out cases and comparing fixed late fusion with zero imputation, rule-based modality-availability routing, learned MoE gating, and task-conditioned MoE. Performance was assessed using accuracy, F1-score, AUROC, receiver operating characteristic (ROC) curves, and confusion matrices. Paired-bootstrap comparisons were used to estimate AUROC gains and 95% confidence intervals (CIs). The mean AUROC comparison across the nine tasks was treated as the primary aggregate comparison, whereas task-specific comparisons were considered secondary and exploratory. Benjamini–Hochberg correction was applied to the nine task-specific P values to control the false-discovery rate. Decision-curve analysis was additionally performed in the held-out test set by comparing task-conditioned MoE, fixed late fusion, treat-all, and treat-none strategies across threshold probabilities from 0.01 to 0.80. Deployability was characterized by model parameters, floating-point operations (FLOPs), latency, throughput, and peak video random-access memory (VRAM) usage under a fixed input setting.

To determine whether OmniBreast provided predictive value beyond routinely available clinical information, we additionally developed endpoint-specific clinical-variable baseline models using multivariable logistic regression and XGBoost. Predictors included age, menopausal status, breast density, mammography view completeness, and histological type. Variables that directly defined or duplicated the target endpoint were excluded from the corresponding model; specifically, TNM components were not used to predict clinical stage, and the corresponding immunohistochemical results were not used to predict ER, PR, HER2, or Ki-67 status. Free-text radiology and pathology reports were not included. The clinical baselines used the same patient-level training, validation, and held-out test partitions as OmniBreast, and AUROC differences were assessed using paired bootstrap resampling.

### 2.4 Cohort derivation, patient-level allocation, and external endpoint-extension cohort

We retrospectively screened 1,428 patients treated at The First Affiliated Hospital of Bengbu Medical University between January 2016 and December 2023. After excluding 174 patients because of unavailable paired radiology–pathology data (n = 86), missing benchmark labels (n = 48), duplicate or repeat records (n = 19), or poor-quality images (n = 21), 1,254 patients underwent further curation; an additional 331 were excluded because of incomplete annotations or quality-control concerns (n = 103), missing preprocessing requirements (n = 74), or failure to meet the final patient-level curation criteria (n = 154), yielding a final internal cohort of 923 patients. Nine supervised tasks were defined, including T, N, and M staging, clinical stage, histological grade, and ER, PR, HER2, and Ki-67 status. The cohort was divided at the patient level into training (n = 646), internal validation (n = 92), and held-out test (n = 185) sets, with the latter reserved exclusively for final evaluation. The complete cohort derivation is shown in Supplementary Figure S1, and endpoint-specific counts for the training, validation, and held-out test partitions are provided in Supplementary Table S1.

At Hunan Cancer Hospital, 394 patients were screened for the independent external endpoint-extension cohort. After excluding 135 patients because mammography or pathology material was unavailable (n = 62), pCR or TNBC endpoint labels were missing (n = 39), duplicate records or repeat admissions were identified (n = 11), or image quality was inadequate (n = 23), 259 patients remained for feasibility testing. For comparison, clinical-variable baseline models were constructed using routinely available clinicopathological variables in this cohort. For the TNBC task, ER, PR, and HER2 were excluded from the predictor set because they served as the reference standard for defining TNBC. The pathology-image AI model was therefore evaluated as an auxiliary prediction model rather than as a replacement for standard immunohistochemistry. All external models were evaluated using AUROC, accuracy, F1-score, sensitivity, and specificity.

### 2.5 Post hoc visualization and clinical-variable contribution analysis

To improve model transparency rather than to infer biological mechanisms, we performed *post hoc* visualization of the radiology branch using gradient-based activation mapping. For representative cases from the held-out set, class-specific response maps were generated from the final convolutional feature representations and normalized to the range of 0–1 for visualization. In addition to continuous heatmaps, thresholded high-response regions were derived to highlight the image areas most strongly associated with the model output. We also performed Shapley additive explanations (SHAP)-based quantitative analysis for the clinical-variable baseline models in the external endpoint-extension cohort. For the mammography-based pCR prediction task, SHAP values were calculated for routinely available clinicopathological variables included in the clinical-variable baseline model. For the pathology-based TNBC prediction task, ER, PR, and HER2 were not included as predictors because they served as the reference standard for defining TNBC. Therefore, SHAP analysis for TNBC prediction was performed using non-definitional clinical variables, including Ki-67 index, histological grade, age, clinical stage, T stage, N stage, postmenopausal status, and invasive ductal type. Mean absolute SHAP values were used to quantify the relative contribution of each clinical variable to baseline predictions.

## 3 Results

### 3.1 Single-modality benchmarking across nine clinical tasks

**FIGURE 2:**
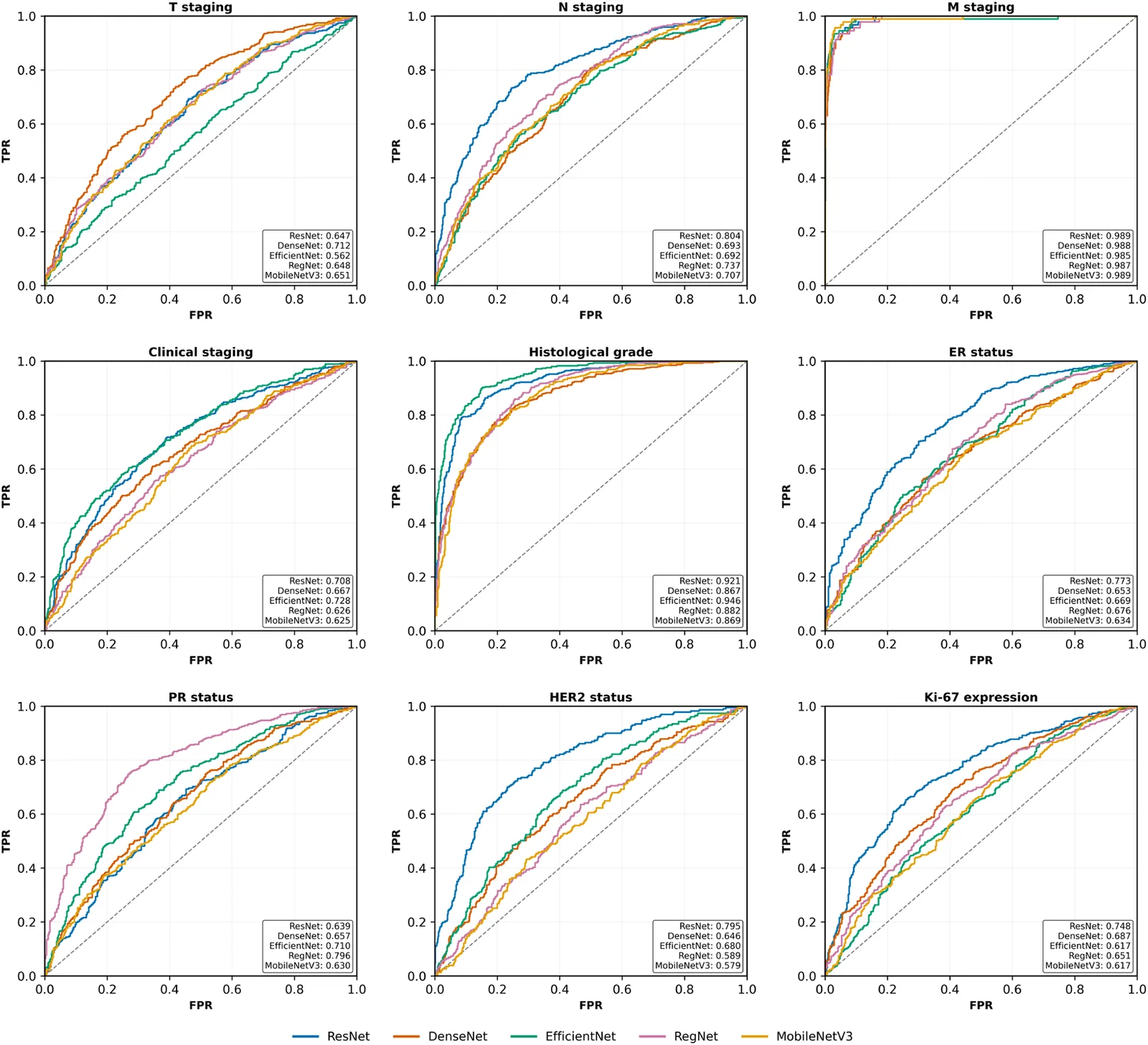
Pathology-only receiver operating characteristic (ROC) curves for nine clinical tasks across five backbones. ROC curves are shown for each endpoint using five pathology CNN backbones. Legends report the pathology AUROC for each backbone; the same estimates are summarized in Figure 3A and Supplementary Table S2. Diagonal lines indicate chance-level performance. Radiology-only AUROCs are reported separately in Figure 3B and Supplementary Table S3.

**FIGURE 3:**
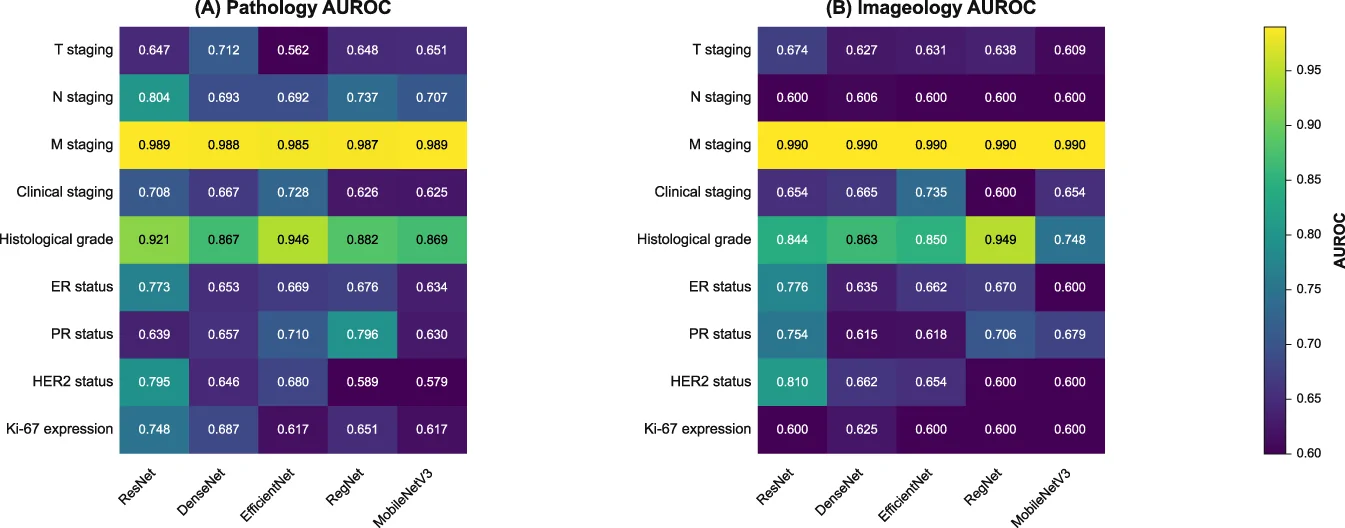
Single-modality performance landscape across tasks and backbones. **(A)** AUROC heatmap for pathology models across nine tasks and five CNN backbones (ResNet, DenseNet, EfficientNet, RegNet and MobileNetV3); the corresponding pathology ROC curves are presented in Figure 2, and the estimates are tabulated in Supplementary Table S2. **(B)** AUROC heatmap for radiology (mammography) models using the same task-backbone grid; the estimates are tabulated in Supplementary Table S3, and radiology ROC curves are not displayed in Figure 2. Each cell reports the AUROC for the corresponding task and backbone.

We established single-modality performance baselines by training five CNN backbones (ResNet, DenseNet, EfficientNet, RegNet and MobileNetV3) on pathology and radiology inputs separately for nine clinically relevant endpoints. Figure 2 displays pathology-only ROC curves, and the corresponding pathology AUROCs are summarized in Figure 3A and Supplementary Table S2. Across the pathology models, AUROCs range from 0.562 to 0.989, with the strongest discrimination observed for M staging and histological grade. Radiology-only AUROCs are presented separately in Figure 3B and Supplementary Table S3 and are not represented in Figure 2. Accuracy results for pathology-only and radiology-only models are summarized in Tables 1, 2.

**TABLE 1:** Pathology-only accuracy across tasks and backbones. Accuracy for pathology-only models across nine tasks, reported for five backbones on the held-out set.

task	ResNet	DenseNet	EfficientNet	RegNet	MobileNetV3
Clinical staging	0.616	0.632	0.616	0.676	0.578
ER status	0.703	0.616	0.638	0.568	0.568
HER2 status	0.741	0.714	0.714	0.741	0.692
Histological grade	0.908	0.816	0.908	0.816	0.816
Ki-67 expression	0.703	0.681	0.589	0.638	0.589
M Staging	0.962	0.978	1.000	0.962	0.978
N staging	0.762	0.659	0.659	0.719	0.659
PR status	0.616	0.589	0.681	0.751	0.589
T Staging	0.600	0.638	0.541	0.622	0.638

**TABLE 2:** Radiology-only accuracy across tasks and backbones. Accuracy for radiology-only models across nine tasks, reported for five backbones on the held-out set.

task	ResNet	DenseNet	EfficientNet	RegNet	MobileNetV3
Clinical staging	0.616	0.616	0.692	0.503	0.616
ER status	0.724	0.589	0.638	0.638	0.546
HER2 status	0.762	0.622	0.622	0.427	0.524
Histological grade	0.816	0.816	0.816	0.908	0.724
Ki-67 expression	0.454	0.589	0.546	0.411	0.362
M Staging	0.962	0.962	0.962	0.962	0.962
N staging	0.481	0.562	0.562	0.562	0.400
PR status	0.724	0.589	0.589	0.681	0.638
T Staging	0.638	0.600	0.600	0.600	0.562

Accuracy patterns largely mirrored AUROC outcomes, while also revealing task-specific calibration characteristics and threshold sensitivity. For instance, pathology models achieved high accuracy for histological grade (0.816–0.908) and M staging (0.962–1.000), whereas radiology models exhibited superior accuracy for clinical staging with EfficientNet (0.692) and comparably high accuracy for M staging (0.962 across all backbones). Collectively, these modality-specific performance heatmaps (Figure 3) provide a concise performance atlas across all endpoints, revealing both backbone-specific performance variability and clinically meaningful performance gaps between modalities.

### 3.2 Modality-specialized “best single” experts reveal complementary strengths

To quantify the complementary strengths of the two modalities more directly, we selected the best-performing backbone for each task within each modality and compared their key operating characteristics (Table 3). Several endpoints were better predicted by pathology models, particularly nodal status and proliferation-related biomarkers: the selected pathology model for N staging (ResNet) reached an AUROC of 0.804 with an accuracy of 0.762, whereas the selected radiology model for the same task (DenseNet) achieved an AUROC of 0.606 with an accuracy of 0.562. A similar performance gap was observed for Ki-67 expression, where pathology models outperformed radiology models (AUROC 0.748 vs 0.625; accuracy 0.703 vs 0.589).

**TABLE 3:** Best single-modality models for each task. For each endpoint and modality (radiology or pathology), the best-performing backbone (selected by validation AUROC) is listed together with held-out accuracy, F1-score, AUROC, sensitivity and specificity, enabling modality-specific benchmarking per clinical task.

task	modality	best_model	accuracy	f1_score	auc	sensitivity	specificity
Clinical staging	Radiology	EfficientNet	0.692	0.678	0.735	0.690	0.694
Clinical staging	Pathology	RegNet	0.676	0.663	0.626	0.678	0.673
ER status	Radiology	ResNet	0.724	0.773	0.776	0.725	0.723
ER status	Pathology	ResNet	0.703	0.753	0.773	0.700	0.708
HER2 status	Radiology	ResNet	0.762	0.676	0.810	0.767	0.760
HER2 status	Pathology	ResNet	0.741	0.647	0.795	0.733	0.744
Histological grade	Radiology	RegNet	0.908	0.908	0.949	0.913	0.903
Histological grade	Pathology	EfficientNet	0.908	0.908	0.946	0.913	0.903
Ki-67 expression	Radiology	DenseNet	0.589	0.616	0.625	0.587	0.593
Ki-67 expression	Pathology	ResNet	0.703	0.726	0.748	0.702	0.704
M Staging	Radiology	ResNet	0.962	0.667	0.990	1.000	0.961
M Staging	Pathology	ResNet	0.962	0.667	0.989	1.000	0.961
N staging	Radiology	DenseNet	0.562	0.497	0.606	0.556	0.566
N staging	Pathology	ResNet	0.762	0.714	0.804	0.764	0.761
PR status	Radiology	ResNet	0.724	0.758	0.754	0.727	0.720
PR status	Pathology	RegNet	0.751	0.783	0.796	0.755	0.747
T Staging	Radiology	ResNet	0.638	0.621	0.674	0.640	0.636
T Staging	Pathology	DenseNet	0.638	0.621	0.712	0.640	0.636

Conversely, several clinically critical endpoints were at least as well captured by radiology models. For HER2 status, the selected radiology expert (ResNet) achieved an AUROC of 0.810 with an accuracy of 0.762, outperforming the selected pathology expert (AUROC 0.795; accuracy 0.741). Radiology models also provided a performance advantage for clinical staging (AUROC 0.735; accuracy 0.692) over pathology models (AUROC 0.626; accuracy 0.676). M staging was well predicted by both modalities (radiology AUROC 0.990 and pathology AUROC 0.989; accuracy 0.962 for both). This finding suggests that for specific labels, either modality can provide predictive signal, and model routing can therefore be driven by data availability and clinical workflow considerations. These findings indicate that backbone performance was endpoint- and modality-dependent. Therefore, task-specific expert selection was more appropriate for the present framework than reliance on a single global backbone.

### 3.3 Multimodal fusion and task-conditioned dynamic routing improve discrimination

**FIGURE 4:**
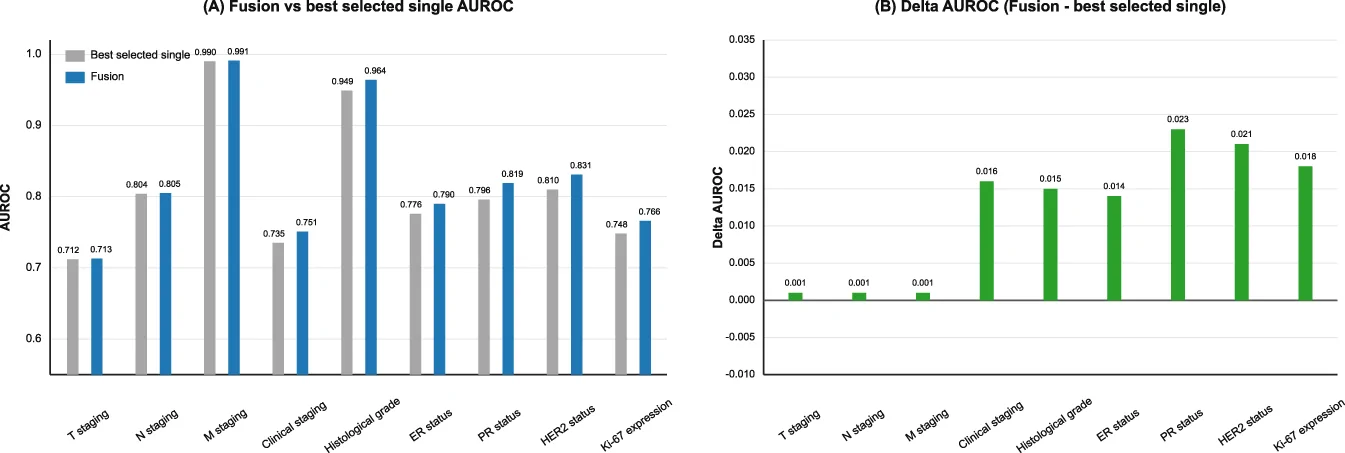
Benefit of multimodal fusion over the best single modality. **(A)** Per-task AUROC comparison between multimodal fusion (late-fusion transformer) and the stronger selected single-modality expert (pathology or radiology) for each endpoint. **(B)** Absolute gain in AUROC (ΔAUROC = Fusion − stronger selected single) across tasks. The plotted estimates and differences are also reported in Table 4 and Supplementary Table S4 and are rounded to three decimal places.

We first evaluated fixed multimodal integration using a late-fusion transformer that fuses modality-specific embeddings from the selected single-modality models (Table 4; Figure 4; Supplementary Table S4). Across the nine clinical tasks, fixed late fusion improved AUROC relative to the stronger selected single-modality expert, with absolute AUROC gains ranging from 0.001 to 0.023 and a mean gain of 0.012. The largest gains were observed for PR status (+0.023), HER2 status (+0.021), Ki-67 expression (+0.018), and clinical staging (+0.016). Mean accuracy increased from 0.767 for the stronger selected single-modality experts to 0.781 for fixed late fusion.

**TABLE 4:** Multimodal fusion versus best single modality. Per-task comparison of multimodal fusion (late-fusion transformer) with the best-performing single-modality model(s). For each task, the table reports: best pathology model and performance, best radiology model and performance, fusion method, fusion accuracy/F1/AUROC, and performance deltas relative to the best single modality (ΔAccuracy and ΔAUROC).

task	pathology_best_model	pathology_acc	pathology_auc	radiology_best_model	radiology_acc	radiology_auc	fusion_method	fusion_accuracy	fusion_f1_score	fusion_auc	delta_acc_vs_best_single	delta_auc_vs_best_single
Clinical staging	RegNet	0.676	0.626	EfficientNet	0.692	0.735	Transformer (late fusion)	0.714	0.667	0.751	0.022	0.016
ER status	ResNet	0.703	0.773	ResNet	0.724	0.776	Transformer (late fusion)	0.746	0.802	0.790	0.022	0.014
HER2 status	ResNet	0.741	0.795	ResNet	0.762	0.810	Transformer (late fusion)	0.773	0.632	0.831	0.011	0.021
Histological grade	EfficientNet	0.908	0.946	RegNet	0.908	0.949	Transformer (late fusion)	0.924	0.925	0.964	0.016	0.015
Ki-67 expression	ResNet	0.703	0.748	DenseNet	0.589	0.625	Transformer (late fusion)	0.714	0.741	0.766	0.011	0.018
M Staging	ResNet	0.962	0.989	ResNet	0.962	0.990	Transformer (late fusion)	0.978	0.778	0.991	0.016	0.001
N staging	ResNet	0.762	0.804	DenseNet	0.562	0.606	Transformer (late fusion)	0.773	0.753	0.805	0.011	0.001
PR status	RegNet	0.751	0.796	ResNet	0.724	0.754	Transformer (late fusion)	0.757	0.791	0.819	0.006	0.023
T Staging	DenseNet	0.638	0.712	ResNet	0.638	0.674	Transformer (late fusion)	0.654	0.670	0.713	0.016	0.001

We then compared fixed late fusion with dynamic routing and MoE baselines (Supplementary Table S5). Learned MoE gating improved mean AUROC to 0.830, and task-conditioned MoE further improved mean AUROC to 0.834, compared with 0.826 for fixed late fusion and 0.813 for the stronger selected single-modality expert. The task-conditioned MoE showed the largest gains over fixed fusion for Ki-67 expression (+0.012), N and T staging (+0.011 each), and HER2 and PR status (+0.010 each). The oracle selector achieved a mean AUROC of 0.854, suggesting that additional headroom remains for more advanced per-case expert selection.

The task-conditioned MoE produced confusion-matrix-based metrics calculated from the 185-patient held-out test set (Supplementary Table S6). For example, it achieved an AUROC of 0.971 and accuracy of 0.930 for histological grade, 0.841 and 0.784 for HER2 status, and 0.816 and 0.778 for N staging. Ablation analysis supported the contribution of both task conditioning and modality-awareness: removing the task embedding reduced mean AUROC from 0.834 to 0.828, while removing the modality mask reduced mean AUROC to 0.826 (Supplementary Table S7). The learned gating weights were clinically plausible, with higher pathology-expert weights for N staging and Ki-67 expression, higher radiology-expert weight for HER2 status, and higher fusion-expert weight for T staging (Supplementary Table S8).

We further compared the task-conditioned model with routinely available clinical-variable baselines. Across the nine primary tasks, logistic regression and XGBoost achieved mean AUROCs of 0.698 and 0.724, respectively, whereas task-conditioned MoE achieved a mean AUROC of 0.834. Compared with the stronger XGBoost baseline, task-conditioned MoE improved mean AUROC by 0.110 (95% CI, 0.091–0.129; P < 0.001) and achieved higher discrimination across all nine endpoints. The largest improvements were observed for histological grade (+0.168), HER2 status (+0.149), M staging (+0.145), and PR status (+0.123; Supplementary Table S9).

### 3.4 Task-level discrimination and error structure across representative endpoints

Missing-modality analysis further supported the value of dynamic routing (Supplementary Table S10). Under 30%, 50%, and 70% random missing-modality settings, task-conditioned MoE achieved mean AUROCs of 0.804, 0.793, and 0.779, respectively, outperforming fixed late fusion with zero imputation by 0.032, 0.051, and 0.067 and outperforming rule-based routing by 0.015, 0.018, and 0.024. Paired-bootstrap analysis showed that fixed late fusion improved mean AUROC over the stronger selected single-modality expert by 0.012 (95% CI, 0.006–0.018; P = 0.008), while task-conditioned MoE provided an additional mean AUROC gain of 0.009 over fixed fusion (95% CI, 0.005–0.013; P = 0.011; Supplementary Table S11). Per-task AUROC differences, 95% confidence intervals, and nominal P values are reported in Supplementary Table S11. For the nine task-conditioned MoE versus fixed-fusion comparisons, none remained significant after Benjamini–Hochberg correction (all adjusted P ≥ 0.072), and these analyses were therefore interpreted as exploratory. Decision-curve analysis further showed that task-conditioned MoE provided only small, task-dependent improvements in net benefit over fixed late fusion within limited threshold ranges, with substantial curve overlap for several endpoints (Supplementary Figure S2). These findings indicate that the AUROC gains were modest but directionally consistent, particularly under missing-modality conditions.

To visualize model operating characteristics beyond scalar performance summaries, we generated held-out ROC curves for each pathology backbone (Figure 2). The corresponding pathology AUROC estimates are summarized in Figure 3A and Supplementary Table S2. Radiology-only results are not plotted in Figure 2 and are summarized separately in Figure 3B and Supplementary Table S3.

### 3.5 Subgroup performance across clinically motivated strata

**FIGURE 5:**
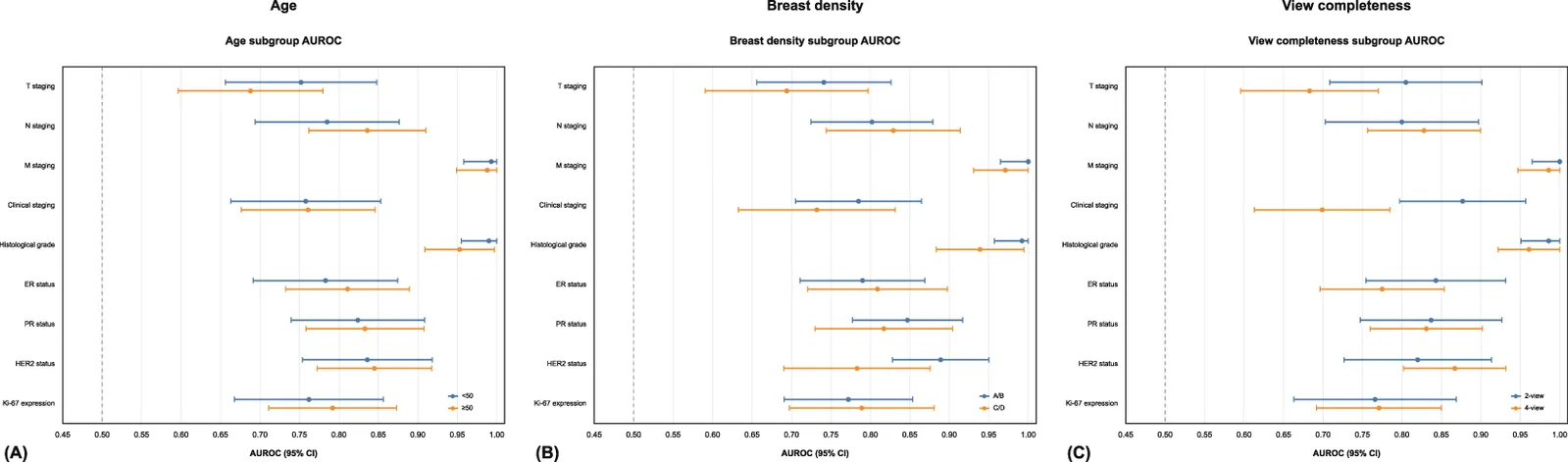
Subgroup performance analysis across prespecified clinical and acquisition strata. Forest plots show subgroup AUROC estimates for each task, stratified by **(A)** age (<50 vs ≥ 50 years), **(B)** breast density (A/B vs C/D), and **(C)** view completeness (2-view vs 4-view). Points show the AUROC estimates reported in Tables 5–13; horizontal bars show descriptive 95% patient-level bootstrap intervals from 2,000 within-subgroup resamples. Subgroup sample sizes are provided in Tables 5–13.

We assessed subgroup performance exclusively in the 185-patient held-out test set, stratified by age (<50 vs ≥ 50 years; n = 78 vs 107), breast density (A/B vs C/D; n = 102 vs 83), and mammography view completeness (2-view vs 4-view; n = 65 vs 120). Accuracy and AUROC were estimated separately within each subgroup (Tables 5–13). Figure 5 displays the subgroup AUROC point estimates with descriptive 95% patient-level bootstrap intervals based on 2,000 within-subgroup resamples. These exploratory estimates should be interpreted cautiously because the subgroup sample sizes, and particularly some endpoint-specific positive counts, were limited.

**TABLE 5:** Subgroup analysis by age: sample sizes. Held-out sample counts per task stratified by age (<50 vs ≥ 50 years).

task	<50	≥50
Clinical staging	78	107
ER status	78	107
HER2 status	78	107
Histological grade	78	107
Ki-67 expression	78	107
M Staging	78	107
N staging	78	107
PR status	78	107
T Staging	78	107

**TABLE 6:** Subgroup analysis by age: accuracy. Held-out accuracy per task stratified by age (<50 vs ≥ 50 years).

task	<50	≥50
Clinical staging	0.718	0.720
ER status	0.731	0.766
HER2 status	0.744	0.813
Histological grade	0.949	0.916
Ki-67 expression	0.718	0.729
M Staging	0.962	0.991
N staging	0.731	0.813
PR status	0.744	0.785
T Staging	0.769	0.589

**TABLE 7:** Subgroup analysis by age: AUROC. Held-out AUROC per task stratified by age (<50 vs ≥ 50 years).

task	<50	≥50
Clinical staging	0.758	0.761
ER status	0.783	0.811
HER2 status	0.836	0.845
Histological grade	0.990	0.953
Ki-67 expression	0.762	0.792
M Staging	0.993	0.988
N staging	0.785	0.836
PR status	0.824	0.833
T Staging	0.752	0.688

Breast-density and view-completeness analyses showed endpoint-specific variation rather than a uniform directional effect (Tables 8–13). Because several subgroup-by-endpoint cells contained relatively few positive cases, particularly for M staging, these analyses were considered exploratory and were not used to support claims of subgroup equivalence.

**TABLE 8:** Subgroup analysis by breast density: sample sizes. Held-out sample counts per task stratified by breast density (A/B vs C/D).

task	A/B	C/D
Clinical staging	102	83
ER status	102	83
HER2 status	102	83
Histological grade	102	83
Ki-67 expression	102	83
M Staging	102	83
N staging	102	83
PR status	102	83
T Staging	102	83

**TABLE 9:** Subgroup analysis by breast density: accuracy. Held-out accuracy per task stratified by breast density (A/B vs C/D).

task	A/B	C/D
Clinical staging	0.745	0.687
ER status	0.745	0.759
HER2 status	0.804	0.759
Histological grade	0.961	0.892
Ki-67 expression	0.725	0.723
M Staging	0.990	0.964
N staging	0.765	0.795
PR status	0.765	0.771
T Staging	0.706	0.614

**TABLE 10:** Subgroup analysis by breast density: AUROC. Held-out AUROC per task stratified by breast density (A/B vs C/D).

task	A/B	C/D
Clinical staging	0.785	0.732
ER status	0.790	0.809
HER2 status	0.889	0.783
Histological grade	0.992	0.939
Ki-67 expression	0.772	0.789
M Staging	1.000	0.971
N staging	0.802	0.829
PR status	0.847	0.817
T Staging	0.741	0.694

**TABLE 11:** Subgroup analysis by view completeness: sample sizes. Held-out sample counts per task stratified by mammography view completeness (2-view vs 4-view).

task	2-view	4-view
Clinical staging	65	120
ER status	65	120
HER2 status	65	120
Histological grade	65	120
Ki-67 expression	65	120
M Staging	65	120
N staging	65	120
PR status	65	120
T Staging	65	120

**TABLE 12:** Subgroup analysis by view completeness: accuracy. Held-out accuracy per task stratified by view completeness (2-view vs 4-view).

task	2-view	4-view
Clinical staging	0.769	0.692
ER status	0.754	0.750
HER2 status	0.815	0.767
Histological grade	0.938	0.925
Ki-67 expression	0.723	0.725
M Staging	1.000	0.967
N staging	0.738	0.800
PR status	0.800	0.750
T Staging	0.708	0.642

**TABLE 13:** Subgroup analysis by view completeness: AUROC. Held-out AUROC per task stratified by view completeness (2-view vs 4-view).

task	2-view	4-view
Clinical staging	0.877	0.699
ER status	0.843	0.775
HER2 status	0.820	0.867
Histological grade	0.986	0.961
Ki-67 expression	0.766	0.771
M Staging	1.000	0.986
N staging	0.800	0.828
PR status	0.837	0.831
T Staging	0.805	0.683

### 3.6 Performance–efficiency trade-offs support deployment-oriented backbone choices

**FIGURE 6:**
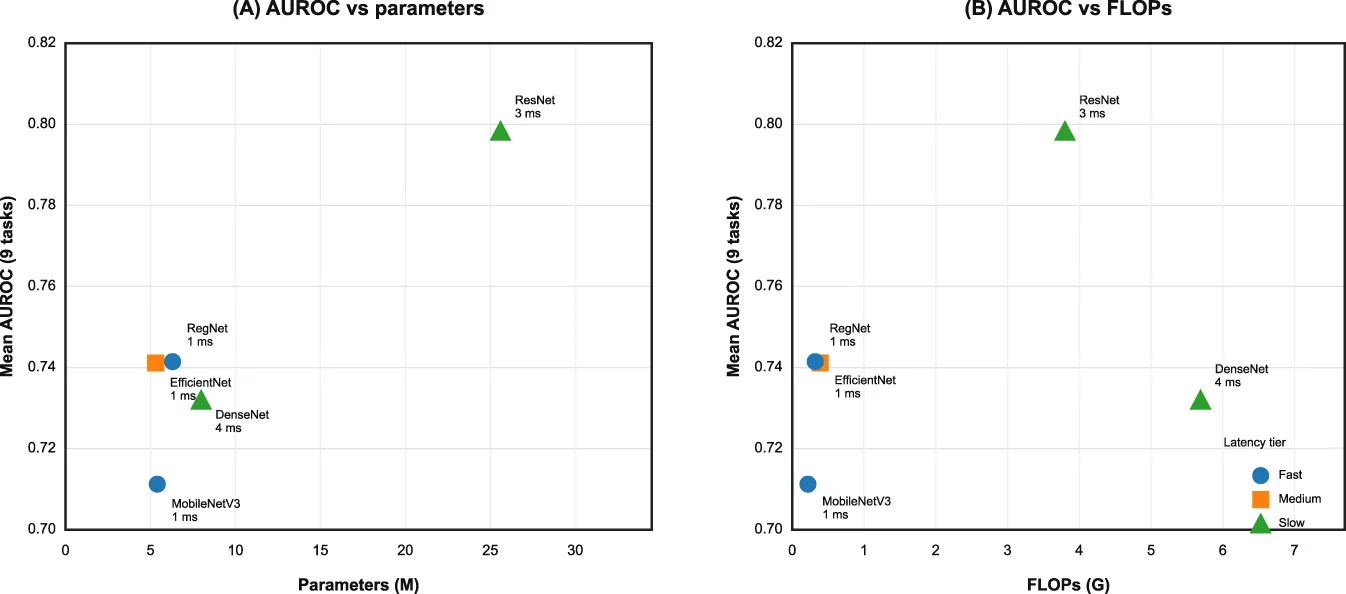
Performance–efficiency trade-off across backbone families. Scatter plots summarize deployability by contrasting predictive discrimination with model complexity. For each backbone, the plotted mean AUROC was calculated by taking the higher of its radiology and pathology AUROC for each task in Figure 3; Supplementary Tables S2, S3 and then averaging across the nine tasks. **(A)** Mean AUROC versus parameter count (M). **(B)** Mean AUROC versus FLOPs **(G)**. Each point corresponds to a backbone family; marker styling groups models into the same latency tiers used throughout the figure. Simulated latency, throughput and VRAM are summarized in Table 14.

Finally, we characterized the deployability of all tested backbones by quantifying model complexity (parameter count and FLOPs) and simulating key inference efficiency metrics (latency, throughput and peak VRAM usage) under a fixed input setting (Table 14; Figure 6). As expected, ResNet50 was the most resource-intensive model (25.6 M parameters, 3.8 GFLOPs; simulated latency 3.197 m), whereas EfficientNetB0, RegNetY-400MF and MobileNetV3-Large clustered into a lightweight model regime (∼5–6 M parameters; ≤0.39 GFLOPs) with substantially lower inference latency (∼0.985–1.078 m) and higher throughput (∼928–1015 fps).

**TABLE 14:** Model complexity and simulated inference efficiency. Parameter count (M) and FLOPs (G) for each backbone, together with simulated inference latency (ms), throughput (fps), and peak VRAM (GB) under a fixed input setting, to contextualize performance-efficiency trade-offs for deployment.

model	params_M	flops_G	latency_ms_simulated	throughput_fps_simulated	vram_GB_simulated
ResNet50	25.6	3.8	3.197	312.8	0.512
DenseNet121	7.98	5.69	4.025	248.4	0.374
EfficientNetB0	5.3	0.39	1.078	927.6	0.241
RegNetY-400MF	6.3	0.32	1.052	950.6	0.249
MobileNetV3-large	5.4	0.219	0.985	1015.2	0.238

When mapping these resource profiles against task performance (Figure 6), the results help explain why lightweight backbones such as EfficientNet, RegNet, and MobileNetV3 offered favorable efficiency profiles, although the best-performing task-specific experts were often ResNet-based in our benchmark (Table 3). In practice, these findings suggest a viable clinical deployment strategy in which the inference workflow can prioritize lightweight backbone experts for high-throughput screening and routine biomarker triage while retaining more computationally intensive models only for specific endpoints or contexts where the incremental performance gains justify the higher resource cost. Additional dynamic-routing baselines introduced only limited computational overhead: task-conditioned MoE required 11.0–51.6 M parameters and 2.20–6.58 m latency, compared with 10.8–51.2 M parameters and 2.10–6.45 m latency for fixed late fusion (Supplementary Table S12). Collectively, these results demonstrate that OmniBreast can be configured not only for predictive performance, but also to align with real-world operational constraints.

### 3.7 External endpoint-extension feasibility analysis for mammography-based pCR and pathology-based TNBC prediction

To assess cross-center feasibility on two clinically relevant endpoint-extension tasks, we performed independent external testing in a cohort of 259 patients from Hunan Cancer Hospital. For mammography-based pCR prediction, the clinical-variable baseline achieved an AUROC of 0.682, an accuracy of 0.660, an F1-score of 0.494, a sensitivity of 0.558, and a specificity of 0.703. In comparison, the mammography-only AI model achieved higher performance, with an AUROC of 0.758, an accuracy of 0.726, an F1-score of 0.590, a sensitivity of 0.662, and a specificity of 0.753.

For pathology-based TNBC prediction, the clinical-variable baseline achieved an AUROC of 0.746, an accuracy of 0.710, an F1-score of 0.607, a sensitivity of 0.659, and a specificity of 0.737. The pathology-only AI model further improved performance, achieving an AUROC of 0.872, an accuracy of 0.807, an F1-score of 0.734, a sensitivity of 0.784, and a specificity of 0.819. These findings indicate that modality-specific AI models provided additional predictive value beyond conventional clinical-variable baselines in the external endpoint-extension cohort. Detailed external comparison results are summarized in Supplementary Tables S13-15.

### 3.8 Post hoc visualization and SHAP-based clinical-variable contribution analysis

**FIGURE 7:**
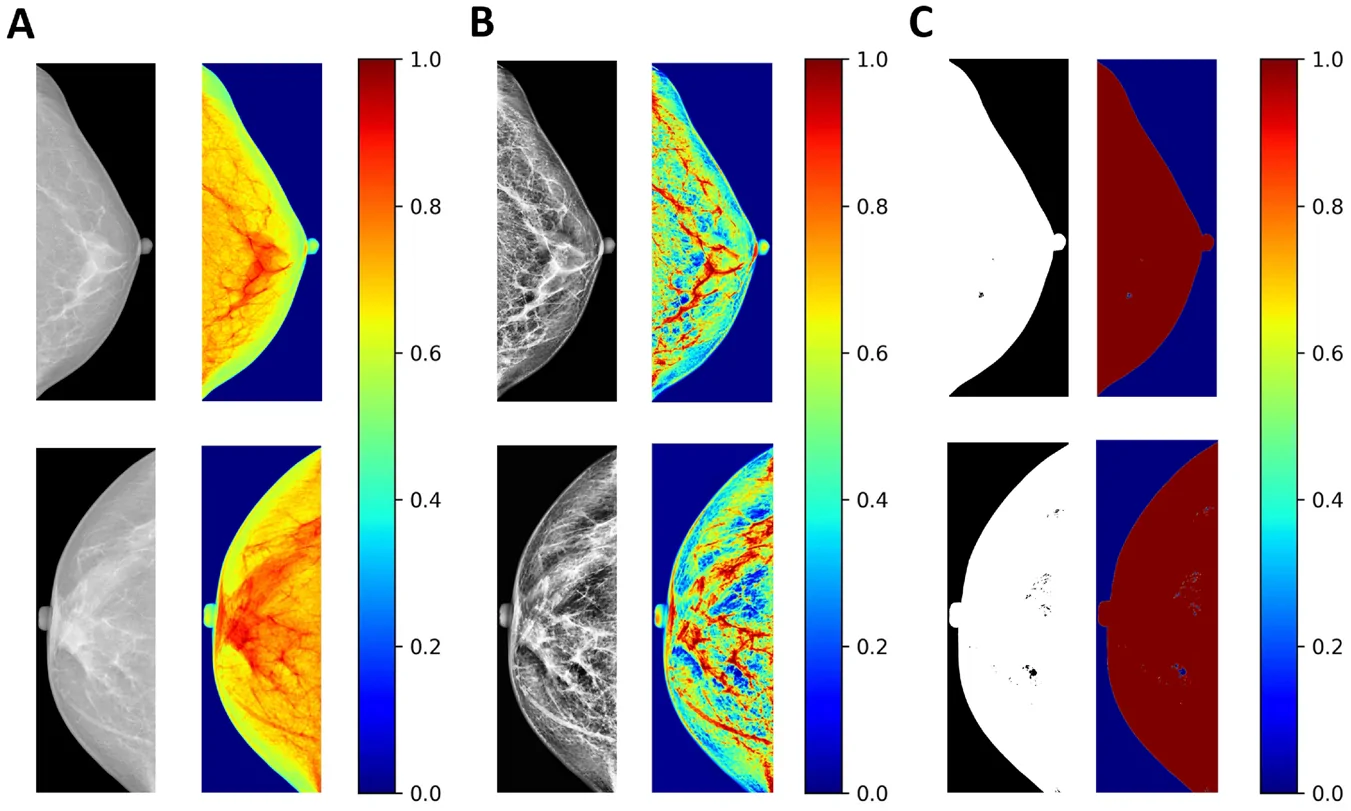
Representative *post hoc* visualization of the radiology branch in OmniBreast. Representative mammography cases from the held-out set are shown to illustrate *post hoc* model visualization. **(A and B)** Original mammograms and the corresponding normalized activation maps (0–1), in which warmer colors indicate stronger model response and greater contribution to the prediction. Panel A shows a relatively diffuse response pattern across the breast parenchyma, whereas panel B demonstrates more heterogeneous and focal activation. **(C)** Thresholded high-response regions derived from the activation maps, shown together with the original mammographic appearance and the corresponding continuous heatmap, highlighting the spatial concentration of the most influential imaging regions within the breast area rather than the background. These visualizations provide qualitative evidence of where the radiology model derives decision-Supplementary Material.

To provide *post hoc* insight into model decision-making, we visualized activation maps for representative mammography cases from the held-out set (Figure 7). The continuous response maps showed that the radiology model primarily focused on intramammary regions with heterogeneous internal structure, whereas background areas contributed minimally to the prediction (Figures 7A,B). After thresholding, the highest-response regions remained spatially confined within the breast area and formed relatively focal patterns rather than diffuse nonspecific activation (Figure 7C). These qualitative findings suggest that the radiology expert responded to structured image regions rather than diffuse background areas, providing *post hoc* evidence for the technical plausibility of the imaging-side outputs of OmniBreast. To complement the qualitative activation-map analysis, we further performed SHAP-based quantitative analysis of the clinical-variable baseline models used in the external endpoint-extension cohort. For mammography-based pCR prediction, the highest mean absolute SHAP values were observed for clinical stage, HER2 status, Ki-67 index, T stage, and N stage, indicating that the clinical baseline mainly relied on established disease-burden and tumor-biology variables. For pathology-based TNBC prediction, ER, PR, and HER2 were excluded because they defined the TNBC label. Among the remaining non-definitional variables, Ki-67 index, histological grade, age, clinical stage, and T stage showed the largest SHAP contributions. These results provide a quantitative explanation of the clinical-variable baselines and help contextualize the additional predictive value of the image-based AI models. The SHAP analysis is shown in Supplementary Figure S3.

## 4 Discussion

OmniBreast provides a task-conditioned multimodal framework that integrates breast cancer staging and biomarker profiling within a single decision pipeline, and our results show that this design yields task-specific gains over strong single-modality baselines. Across nine clinically relevant tasks spanning TNM components, clinical stage, histological grade, and key immunohistochemical surrogates (ER/PR/HER2/Ki-67), single-modality models achieved task-dependent but clinically informative performance, with clear modality-dependent strengths. Pathology tended to be more informative for nodal status and proliferative activity, aligning with established deep learning benchmarks in computational pathology (Ehteshami Bejnordi et al., 2017; Couture et al., 2018), whereas radiology contributed stronger cues for HER2 and clinical staging, highlighting that the best modality is endpoint-specific rather than universal (McKinney et al., 2020). Fixed multimodal fusion improved or matched AUROC relative to the best single-modality expert, and the additional dynamic-routing experiments showed that learned MoE gating and task-conditioned MoE provided further modest gains. This finding is consistent with recent work in oncology showing that radiomics and pathomics can capture complementary cross-scale information (Lipkova et al., 2022; Chen et al., 2022), but the magnitude of improvement should be interpreted as incremental rather than definitive evidence of methodological superiority. Importantly, task-conditioned OmniBreast also outperformed the logistic-regression and XGBoost clinical-variable baselines across all nine tasks, suggesting that the radiology and pathology representations provided complementary discrimination beyond the routinely available non-definitional variables included in these models.

The contribution of OmniBreast should therefore be interpreted as a clinically structured radiology-pathology benchmark and task-conditioned routing workflow rather than a general-purpose medical AI agent. Compared with previous multimodal breast cancer AI studies that primarily addressed individual endpoints such as treatment response, pathological complete response, recurrence risk, or molecular subtype prediction, OmniBreast jointly evaluated nine clinically relevant staging and biomarker tasks. In addition, it benchmarked five backbone networks within each modality, evaluated fixed late fusion, learned MoE gating, task-conditioned MoE routing, ablated task embeddings and modality masks, and tested missing-modality robustness. This design provides methodological flexibility and practical compatibility with real-world clinical workflows, where different patients may have different combinations of imaging and pathology data.

Two design features may explain the observed benefit of OmniBreast. First, the framework decouples modality-specific representation learning from downstream fusion, allowing each backbone to serve as an expert optimized for a specific label under a specific modality. This decomposition may reduce negative transfer, a phenomenon in which competing objectives in multi-task learning degrade performance on individual tasks (Crawshaw, 2020; Fifty et al., 2021). Second, the task-conditioned MoE gate uses task embeddings and modality-availability masks to learn endpoint-specific expert weights. The resulting gating patterns were clinically plausible: pathology weights were highest for N staging and Ki-67 expression, radiology weight was highest for HER2 status, and fusion weight was highest for T staging. These results suggest that task information and modality availability both contributed to the learned routing behavior, although the gain over fixed late fusion remained modest.

It is important to distinguish this implementation from autonomous medical AI agents. OmniBreast does not perform open-ended planning, tool use, free-form multi-agent communication, or autonomous clinical decision-making. Instead, it implements a bounded dynamic routing mechanism in which the learned MoE gate assigns weights to predefined radiology, pathology, and fusion experts. Therefore, our claim is limited to task-conditioned expert routing within a supervised multimodal prediction framework. The task-conditioned MoE improved mean AUROC by 0.009 over fixed late fusion and showed larger relative gains under simulated missing-modality conditions, suggesting practical value in incomplete-data workflows without implying superiority over all existing agentic or general dynamic routing architectures.

Beyond predictive accuracy, OmniBreast has practical implications for how AI could be introduced into breast cancer workflows. The *post hoc* activation maps suggested that the radiology branch primarily responded to anatomically meaningful breast regions rather than background areas, supporting the technical plausibility of the learned imaging representations. The SHAP-based analysis of the clinical-variable baseline models provided a quantitative interpretability reference. This analysis identified clinically plausible contributors to baseline prediction, including stage-related variables, receptor status, Ki-67 index, and histological grade, and helped contextualize the incremental value of image-based AI models beyond conventional clinical information. At the same time, these *post hoc* explanations should be interpreted cautiously because activation maps and SHAP values provide attribution-based evidence rather than direct lesion-level annotation, causal localization, or biological mechanism. A unified framework spanning screening-adjacent triage, staging refinement, and biomarker profiling may reduce fragmentation across tools and provide consistent adjunctive decision support across the patient journey (Kline et al., 2022; Colen and Piwnica-Worms, 2016; Topol, 2019). The performance-efficiency trade-off analysis suggests that such a system can be configured for operational constraints: lightweight backbones (e.g., MobileNetV3/EfficientNet (Tan and Le, 2019)) offer favorable deployment characteristics while remaining competitive, supporting high-throughput use cases. Meanwhile, the fusion pathway offers a principled mechanism to escalate to more comprehensive inference when multi-source data are available. Subgroup analyses across age, density, and view completeness did not reveal systematic performance degradation across these prespecified strata, although endpoint-specific heterogeneity remained, a prerequisite for translation discussed extensively in recent deployment guidelines (Kelly et al., 2019). In aggregate, these findings provide a practical basis for scalable, workflow-aware multimodal AI that can be extended to incorporate additional endpoints (e.g., treatment response or recurrence risk) as data mature (Kather et al., 2019).

Several limitations should be acknowledged. First, although the current cohort is substantial, broader generalizability will ultimately require multi-center validation across institutions to quantify distribution shift caused by different imaging vendors and staining protocols (Zech et al., 2018). Second, OmniBreast has not yet been packaged as a deployable clinical system. As noted in the CONSORT-AI and SPIRIT-AI reporting guidelines, prospective studies will be needed to evaluate clinical utility, including how the system changes decisions or improves outcomes, beyond retrospective discrimination metrics (Liu et al., 2020; Varoquaux and Cheplygina, 2022). Third, survival-related endpoints were not included in the present study because longitudinal follow-up data are still being collected, and future studies will extend OmniBreast to recurrence and survival prediction once mature outcome data become available. Fourth, although we added learned MoE and task-conditioned routing baselines, these comparisons were conducted within the present retrospective benchmark and did not exhaustively cover all recently proposed agentic, MoE, or dynamic multimodal learning architectures. Future work will therefore focus on scaling to multi-center datasets, incorporating longitudinal outcome endpoints, prospectively evaluating clinical utility, and comparing the framework with broader learned routing and multi-agent baselines.

The external analyses should be interpreted as endpoint-extension tests rather than external validation of all nine primary tasks. In addition, the clinical-variable baseline comparisons were retrospective and require independent replication with prespecified baseline models and standardized clinical inputs. Standardized, independent, and blinded radiologist and pathologist assessments were unavailable for this retrospective cohort; therefore, a formal reader study could not be performed. Report-based models were also not evaluated because routine reports contained information used to establish several reference labels and could consequently introduce target leakage. Prospective reader studies will be required to compare OmniBreast directly with specialist assessment.

## 5 Conclusion

We present OmniBreast, a task-conditioned multimodal AI framework for breast cancer assessment across tumor staging and biomarker profiling. Across nine clinical tasks, performance was endpoint-dependent, and fixed multimodal fusion improved discrimination for most tasks. The high internal M-stage performance was considered exploratory and requires independent validation because distant metastatic disease was not directly represented in the mammographic or H&E inputs. Learned MoE gating and task-conditioned MoE provided additional modest AUROC gains over fixed late fusion and improved robustness under simulated missing-modality settings. The external analyses provided preliminary evidence of feasibility for two related modality-specific endpoint-extension tasks but did not externally validate the nine primary staging and biomarker tasks. Accordingly, the generalizability of OmniBreast across the primary tasks remains to be established through multicenter external validation and prospective clinical evaluation.

## Data Availability

The original contributions presented in the study are included in the article/Supplementary Material, further inquiries can be directed to the corresponding authors.
