# Supplementary material for: Development and internal validation of a task-conditioned multimodal radiology–pathology model for breast cancer staging and biomarker profiling: a retrospective cohort study
Source: Front Cell Dev Biol. 2026 Aug 21;14:1863777. doi: 10.3389/fcell.2026.1863777 (PMC13541708; doi:10.3389/fcell.2026.1863777)
Supplement: Supplementary file 1 [file DataSheet1.docx]

Supplementary Material

Figure S1. Study flow diagram for cohort derivation and patient-level dataset allocation.

Figure S2. Decision-curve analysis of fixed late fusion and task-conditioned mixture-of-experts models across the nine primary tasks; legend AUROCs correspond to Tables S4 and S5.

Figure S3. SHAP-based quantitative analysis of clinical-variable baseline models.

(A) Mean absolute SHAP values for the clinical-variable baseline model in mammography-based pCR prediction. Clinical stage, HER2 status, Ki-67 index, T stage, and N stage showed the largest contributions to baseline prediction.(B) SHAP summary plot for the pCR clinical-variable baseline model. Each point represents one patient-level observation, and color indicates the normalized feature value.(C) Mean absolute SHAP values for the clinical-variable baseline model in pathology-based TNBC prediction. ER, PR, and HER2 were not included as predictors because they served as the reference standard for defining TNBC. Ki-67 index, histological grade, age, clinical stage, and T stage showed the largest contributions among non-definitional variables.(D) SHAP summary plot for the TNBC clinical-variable baseline model. This analysis complements heatmap-based visualization by providing quantitative clinical-variable attribution and by offering an interpretable comparator for the image-based AI models.

Table S1. Endpoint-specific sample size and class distribution by patient-level data partition.

| Task | Partition | Total n | Positive n | Negative n | Missing n | Prevalence (%) |
| --- | --- | --- | --- | --- | --- | --- |
| Clinical staging | train | 646 | 304 | 342 | 0 | 47.1 |
| Clinical staging | validation | 92 | 43 | 49 | 0 | 46.7 |
| Clinical staging | test | 185 | 87 | 98 | 0 | 47.0 |
| ER status | train | 646 | 419 | 227 | 0 | 64.9 |
| ER status | validation | 92 | 60 | 32 | 0 | 65.2 |
| ER status | test | 185 | 120 | 65 | 0 | 64.9 |
| HER2 status | train | 646 | 210 | 436 | 0 | 32.5 |
| HER2 status | validation | 92 | 30 | 62 | 0 | 32.6 |
| HER2 status | test | 185 | 60 | 125 | 0 | 32.4 |
| Histological grade | train | 646 | 321 | 325 | 0 | 49.7 |
| Histological grade | validation | 92 | 46 | 46 | 0 | 50.0 |
| Histological grade | test | 185 | 92 | 93 | 0 | 49.7 |
| Ki-67 expression | train | 646 | 363 | 283 | 0 | 56.2 |
| Ki-67 expression | validation | 92 | 52 | 40 | 0 | 56.5 |
| Ki-67 expression | test | 185 | 104 | 81 | 0 | 56.2 |
| M staging | train | 646 | 24 | 622 | 0 | 3.7 |
| M staging | validation | 92 | 3 | 89 | 0 | 3.3 |
| M staging | test | 185 | 7 | 178 | 0 | 3.8 |
| N staging | train | 646 | 251 | 395 | 0 | 38.9 |
| N staging | validation | 92 | 36 | 56 | 0 | 39.1 |
| N staging | test | 185 | 72 | 113 | 0 | 38.9 |
| PR status | train | 646 | 384 | 262 | 0 | 59.4 |
| PR status | validation | 92 | 55 | 37 | 0 | 59.8 |
| PR status | test | 185 | 110 | 75 | 0 | 59.5 |
| T staging | train | 646 | 300 | 346 | 0 | 46.4 |
| T staging | validation | 92 | 43 | 49 | 0 | 46.7 |
| T staging | test | 185 | 86 | 99 | 0 | 46.5 |

Table S2. Pathology AUROC across tasks and CNN backbones.

Pathology-only AUROCs across nine tasks and five backbones. Figure 2A presents these estimates as a heatmap, and Figure 4 presents the corresponding pathology ROC curves.

| task | ResNet | DenseNet | EfficientNet | RegNet | MobileNetV3 |
| --- | --- | --- | --- | --- | --- |
| Clinical staging | 0.708 | 0.667 | 0.728 | 0.626 | 0.625 |
| ER status | 0.773 | 0.653 | 0.669 | 0.676 | 0.634 |
| HER2 status | 0.795 | 0.646 | 0.680 | 0.589 | 0.579 |
| Histological grade | 0.921 | 0.867 | 0.946 | 0.882 | 0.869 |
| Ki-67 expression | 0.748 | 0.687 | 0.617 | 0.651 | 0.617 |
| M staging | 0.989 | 0.988 | 0.985 | 0.987 | 0.989 |
| N staging | 0.804 | 0.693 | 0.692 | 0.737 | 0.707 |
| PR status | 0.639 | 0.657 | 0.710 | 0.796 | 0.630 |
| T staging | 0.647 | 0.712 | 0.562 | 0.648 | 0.651 |

Table S3. Radiology (mammography) AUROC across tasks and CNN backbones.

Radiology-only AUROCs across nine tasks and five backbones. These values correspond to Figure 2B and are not represented in Figure 4, which displays pathology-only ROC curves.

| task | ResNet | DenseNet | EfficientNet | RegNet | MobileNetV3 |
| --- | --- | --- | --- | --- | --- |
| Clinical staging | 0.654 | 0.665 | 0.735 | 0.6 | 0.654 |
| ER status | 0.776 | 0.635 | 0.662 | 0.67 | 0.6 |
| HER2 status | 0.81 | 0.662 | 0.654 | 0.6 | 0.6 |
| Histological grade | 0.844 | 0.863 | 0.85 | 0.949 | 0.748 |
| Ki-67 expression | 0.6 | 0.625 | 0.6 | 0.6 | 0.6 |
| M staging | 0.99 | 0.99 | 0.99 | 0.99 | 0.99 |
| N staging | 0.6 | 0.606 | 0.6 | 0.6 | 0.6 |
| PR status | 0.754 | 0.615 | 0.618 | 0.706 | 0.679 |
| T staging | 0.674 | 0.627 | 0.631 | 0.638 | 0.609 |

Table S4. Best single-modality expert versus fixed late fusion across nine primary tasks.

| Task | Best single AUROC | Best single accuracy | Fixed late-fusion AUROC | Fixed late-fusion accuracy | Delta AUROC | Delta accuracy |
| --- | --- | --- | --- | --- | --- | --- |
| Clinical staging | 0.735 | 0.692 | 0.751 | 0.714 | 0.016 | 0.022 |
| ER status | 0.776 | 0.724 | 0.790 | 0.746 | 0.014 | 0.022 |
| HER2 status | 0.810 | 0.762 | 0.831 | 0.773 | 0.021 | 0.011 |
| Histological grade | 0.949 | 0.908 | 0.964 | 0.924 | 0.015 | 0.016 |
| Ki-67 expression | 0.748 | 0.703 | 0.766 | 0.714 | 0.018 | 0.011 |
| M staging | 0.990 | 0.962 | 0.991 | 0.978 | 0.001 | 0.016 |
| N staging | 0.804 | 0.762 | 0.805 | 0.773 | 0.001 | 0.011 |
| PR status | 0.796 | 0.751 | 0.819 | 0.757 | 0.023 | 0.006 |
| T staging | 0.712 | 0.638 | 0.713 | 0.654 | 0.001 | 0.016 |
| Mean | 0.813 | 0.767 | 0.826 | 0.781 | 0.012 | 0.014 |

Table S5. Comparison with dynamic routing and mixture-of-experts baselines across nine primary tasks.

Learned MoE gating and task-conditioned MoE were compared with best single-modality experts, fixed late fusion, and a non-deployable oracle selector upper bound.

| Task | Best single AUROC | Fixed late fusion AUROC | Learned MoE gating AUROC | Task-conditioned MoE AUROC | Oracle selector AUROC | Delta vs fixed fusion | Delta vs best single |
| --- | --- | --- | --- | --- | --- | --- | --- |
| Clinical staging | 0.735 | 0.751 | 0.756 | 0.759 | 0.784 | 0.008 | 0.024 |
| ER status | 0.776 | 0.790 | 0.794 | 0.798 | 0.820 | 0.008 | 0.022 |
| HER2 status | 0.810 | 0.831 | 0.837 | 0.841 | 0.858 | 0.010 | 0.031 |
| Histological grade | 0.949 | 0.964 | 0.967 | 0.971 | 0.975 | 0.007 | 0.022 |
| Ki-67 expression | 0.748 | 0.766 | 0.774 | 0.778 | 0.805 | 0.012 | 0.030 |
| M staging | 0.990 | 0.991 | 0.990 | 0.991 | 0.992 | 0.000 | 0.001 |
| N staging | 0.804 | 0.805 | 0.812 | 0.816 | 0.846 | 0.011 | 0.012 |
| PR status | 0.796 | 0.819 | 0.824 | 0.829 | 0.852 | 0.010 | 0.033 |
| T staging | 0.712 | 0.713 | 0.717 | 0.724 | 0.758 | 0.011 | 0.012 |
| Mean | 0.813 | 0.826 | 0.830 | 0.834 | 0.854 | 0.009 | 0.021 |

Table S6. Operating-point performance of the task-conditioned MoE in the held-out test set (n = 185).

| Task | AUROC | Accuracy | F1 | Sensitivity | Specificity | PPV | NPV |
| --- | --- | --- | --- | --- | --- | --- | --- |
| Clinical staging | 0.759 | 0.719 | 0.701 | 0.701 | 0.735 | 0.701 | 0.735 |
| ER status | 0.798 | 0.751 | 0.800 | 0.767 | 0.723 | 0.836 | 0.627 |
| HER2 status | 0.841 | 0.784 | 0.697 | 0.767 | 0.792 | 0.639 | 0.876 |
| Histological grade | 0.971 | 0.930 | 0.930 | 0.935 | 0.925 | 0.925 | 0.935 |
| Ki-67 expression | 0.778 | 0.724 | 0.751 | 0.740 | 0.704 | 0.762 | 0.679 |
| M staging | 0.991 | 0.978 | 0.750 | 0.857 | 0.983 | 0.667 | 0.994 |
| N staging | 0.816 | 0.778 | 0.732 | 0.778 | 0.779 | 0.691 | 0.846 |
| PR status | 0.829 | 0.768 | 0.802 | 0.791 | 0.733 | 0.813 | 0.705 |
| T staging | 0.724 | 0.665 | 0.648 | 0.663 | 0.667 | 0.633 | 0.695 |

Table S7. Ablation of task-conditioned dynamic routing.

| Task | MoE without task embedding AUROC | MoE without modality mask AUROC | Full task-conditioned MoE AUROC | Gain from task embedding | Gain from modality mask |
| --- | --- | --- | --- | --- | --- |
| Clinical staging | 0.754 | 0.752 | 0.759 | 0.005 | 0.007 |
| ER status | 0.791 | 0.790 | 0.798 | 0.007 | 0.008 |
| HER2 status | 0.835 | 0.832 | 0.841 | 0.006 | 0.009 |
| Histological grade | 0.966 | 0.965 | 0.971 | 0.005 | 0.006 |
| Ki-67 expression | 0.771 | 0.769 | 0.778 | 0.007 | 0.009 |
| M staging | 0.990 | 0.989 | 0.991 | 0.001 | 0.002 |
| N staging | 0.809 | 0.807 | 0.816 | 0.007 | 0.009 |
| PR status | 0.821 | 0.818 | 0.829 | 0.008 | 0.011 |
| T staging | 0.715 | 0.713 | 0.724 | 0.009 | 0.011 |
| Mean | 0.828 | 0.826 | 0.834 | 0.006 | 0.008 |

Table S8. Mean gating weights for task-conditioned MoE across tasks.

| Task | Radiology expert weight | Pathology expert weight | Fusion expert weight | Weight sum |
| --- | --- | --- | --- | --- |
| Clinical staging | 0.420 | 0.250 | 0.330 | 1.000 |
| ER status | 0.380 | 0.280 | 0.340 | 1.000 |
| HER2 status | 0.550 | 0.180 | 0.270 | 1.000 |
| Histological grade | 0.320 | 0.330 | 0.350 | 1.000 |
| Ki-67 expression | 0.140 | 0.610 | 0.250 | 1.000 |
| M staging | 0.340 | 0.340 | 0.320 | 1.000 |
| N staging | 0.120 | 0.630 | 0.250 | 1.000 |
| PR status | 0.300 | 0.390 | 0.310 | 1.000 |
| T staging | 0.280 | 0.250 | 0.470 | 1.000 |

Table S9. Comparison of clinical-variable baselines and task-conditioned OmniBreast across the nine primary tasks

| Task | Clinical LR AUROC | Clinical XGBoost AUROC | Task-conditioned MoE AUROC | ΔAUROC vs XGBoost | 95% CI | P value |
| --- | --- | --- | --- | --- | --- | --- |
| Clinical stage | 0.681 | 0.704 | 0.759 | +0.055 | 0.006–0.106 | 0.028 |
| ER status | 0.698 | 0.721 | 0.798 | +0.077 | 0.025–0.129 | 0.004 |
| HER2 status | 0.665 | 0.692 | 0.841 | +0.149 | 0.094–0.206 | <0.001 |
| Histological grade | 0.775 | 0.803 | 0.971 | +0.168 | 0.118–0.214 | <0.001 |
| Ki-67 expression | 0.659 | 0.684 | 0.778 | +0.094 | 0.037–0.153 | 0.002 |
| M staging | 0.812 | 0.846 | 0.991 | +0.145 | 0.018–0.270 | 0.029 |
| N staging | 0.689 | 0.711 | 0.816 | +0.105 | 0.046–0.166 | 0.001 |
| PR status | 0.681 | 0.706 | 0.829 | +0.123 | 0.066–0.180 | <0.001 |
| T staging | 0.624 | 0.648 | 0.724 | +0.076 | 0.018–0.134 | 0.011 |
| Mean | 0.698 | 0.724 | 0.834 | +0.110 | 0.091–0.129 | <0.001 |

Table S10. Missing-modality robustness analysis using mean AUROC across nine tasks.

| Missing-modality setting | Fixed late fusion with zero imputation | Rule-based modality-availability routing | Learned MoE gating | Task-conditioned MoE | Task-MoE gain vs fixed fusion | Task-MoE gain vs rule-based |
| --- | --- | --- | --- | --- | --- | --- |
| 0% missing | 0.826 | 0.826 | 0.830 | 0.834 | 0.009 | 0.009 |
| 30% random missing | 0.772 | 0.789 | 0.797 | 0.804 | 0.032 | 0.015 |
| 50% random missing | 0.742 | 0.775 | 0.784 | 0.793 | 0.051 | 0.018 |
| 70% random missing | 0.712 | 0.755 | 0.768 | 0.779 | 0.067 | 0.024 |

Table S11. Paired-bootstrap comparison of AUROC gains; point estimates correspond to the displayed AUROCs in Tables S4 and S5.

| Task | Fixed fusion vs best single: Delta AUROC | 95% CI | P value | Task-conditioned MoE vs fixed fusion: Delta AUROC | 95% CI | P value |
| --- | --- | --- | --- | --- | --- | --- |
| Clinical staging | 0.016 | 0.003 to 0.027 | 0.014 | 0.008 | 0.000 to 0.017 | 0.048 |
| ER status | 0.014 | 0.003 to 0.027 | 0.031 | 0.008 | -0.002 to 0.019 | 0.067 |
| HER2 status | 0.021 | 0.006 to 0.035 | 0.009 | 0.010 | 0.001 to 0.021 | 0.041 |
| Histological grade | 0.015 | 0.005 to 0.026 | 0.018 | 0.007 | -0.001 to 0.014 | 0.122 |
| Ki-67 expression | 0.018 | 0.007 to 0.033 | 0.044 | 0.012 | 0.001 to 0.025 | 0.029 |
| M staging | 0.001 | -0.001 to 0.003 | 0.500 | 0.000 | 0.000 to 0.000 | 1.000 |
| N staging | 0.001 | -0.014 to 0.019 | 0.900 | 0.011 | 0.001 to 0.024 | 0.038 |
| PR status | 0.023 | 0.006 to 0.041 | 0.006 | 0.010 | 0.001 to 0.024 | 0.035 |
| T staging | 0.001 | -0.017 to 0.019 | 0.900 | 0.011 | 0.002 to 0.022 | 0.016 |
| Mean | 0.012 | 0.006 to 0.018 | 0.008 | 0.009 | 0.005 to 0.013 | 0.011 |

Table S12. Deployment overhead of fixed fusion and dynamic routing baselines.

| Model | Components | Routing type | Parameters (M) | FLOPs (G) | Latency (ms) |
| --- | --- | --- | --- | --- | --- |
| Best single-modality expert | 1 selected CNN backbone | N/A | 5.4-25.6 | 0.22-3.80 | 0.98-3.20 |
| Fixed late fusion | Two selected CNN backbones + lightweight transformer | Fixed when both modalities available | 10.8-51.2 | 0.44-7.60 | 2.10-6.45 |
| Rule-based routing workflow | Selected single or fusion model | Manual task/modality rule | 5.4-51.2 | 0.22-7.60 | 0.98-6.45 |
| Learned MoE gating | Radiology/pathology/fusion experts + small MLP gate | Learned modality-level weights | 10.9-51.4 | 0.45-7.63 | 2.16-6.52 |
| Task-conditioned MoE | MoE gate + task embedding + modality mask | Learned task-conditioned weights | 11.0-51.6 | 0.46-7.66 | 2.20-6.58 |
| Oracle selector | Post hoc upper-bound selector | Uses test labels; not deployable | N/A | N/A | N/A |

Table S13. External endpoint-extension feasibility performance for mammography-based pCR prediction and pathology-based TNBC prediction.

| Task | Modality | External n | Positive n (%) | Suggested model | AUROC (95% CI) | Accuracy | F1-score | Sensitivity | Specificity |
| --- | --- | --- | --- | --- | --- | --- | --- | --- | --- |
| pCR prediction | Mammography | 259 | 77 (29.7%) | EfficientNet-B0 | 0.758 (0.698–0.813) | 0.726 | 0.590 | 0.662 | 0.753 |
| TNBC prediction | Pathology | 259 | 88 (34.0%) | ResNet50 | 0.872 (0.830–0.910) | 0.807 | 0.734 | 0.784 | 0.819 |

Table S14. Additional external endpoint-extension feasibility metrics for mammography-based pCR prediction and pathology-based TNBC prediction.

| Task | PPV | NPV | AUPRC |
| --- | --- | --- | --- |
| pCR prediction | 0.531 | 0.840 | 0.552 |
| TNBC prediction | 0.690 | 0.881 | 0.803 |

Table S15. Comparison between modality-specific AI models and clinical-variable baselines in the external endpoint-extension cohort.

| Task | External n | Model / baseline | Input | AUROC | Accuracy | F1-score | Sensitivity | Specificity |
| --- | --- | --- | --- | --- | --- | --- | --- | --- |
| pCR prediction | 259 | Clinical-variable baseline | Clinical variables | 0.682 | 0.660 | 0.494 | 0.558 | 0.703 |
| pCR prediction | 259 | Mammography-only AI model | Mammography | 0.758 | 0.726 | 0.590 | 0.662 | 0.753 |
| TNBC prediction | 259 | Clinical-variable baseline | Clinical variables | 0.746 | 0.710 | 0.607 | 0.659 | 0.737 |
| TNBC prediction | 259 | Pathology-only AI model | Pathology image | 0.872 | 0.807 | 0.734 | 0.784 | 0.819 |
